# The Book World of Medicine and Science

**Published:** 1899-07-22

**Authors:** 


### The Book World of Medicine and Science.

The Elements of Vital Statistics. By Arthur Newsholme, M.D.Lond., F.R.C.P. Third Edition, almost entirely rewritten. (London: Swan Sonnenschein and Co. 1899. Price 7s. 6d.)

According to some people a third edition of a book hardly requires much notice at the hands of a reviewer, but when an author tells us that his work has been almost entirely rewritten we feel that we may fairly take it up again *de novo.* To many minds statistics have no attractions, and pages of tables are repulsive as well as meaningless, nevertheless some knowledge of the manner in which figures may be arranged so as to convey truth, and of the modes in which they may be manipulated so as to perpetuate error, is absolutely necessary to all who presume, or are compelled by their position, to form opinions on public health questions. It has often been slightingly said that statistics may be made to prove anything, and no doubt they may be twisted in such a fashion as to make it very difficult for any who have not studied the subject to detect the fallacies involved in their abuse. But this makes it all the more essential that the subject should be studied, and for this purpose we can have no hesitation in recommending the book before us, for it not only is correct in its principles and accurate in its formulæ throughout, but is interesting and full of information. One of the difficulties which always stands in the way of the statistician is that which arises from the errors which creep into the very facts themselves on which his statistics are founded. A good instance of this is to be found in Dr. Newsholme's first chapter, where he shows by a curious diagram how great are the errors which are liable to affect a mere tabulation of the age distribution of the population, in consequence of those who are careless or uncertain on the subject of their age falling back upon the nearest ten in stating it in figures. Instead of the diagram which represents the number living at each age showing a steady descent from the earliest to the latest periods, it shows a series of peaks, the number of people who say that they are thirty, forty, fifty, or sixty years of age being far in excess of those who give the intermediate years. It is needless to say how an error of fact like this tends to permeate all matters in which the question of age arises. Much has been made of late, especially by Dr. Hope, of Liverpool, of the erroneous estimates of the population made by the Registrar-General during the years which intervene between one census and another; but although Dr. Newsholme admits the justice of the criticisms, and indeed, gives striking instances of the errors entailed by the official method, yet he says that this seems, under present conditions, to be the "least unsatisfactory" basis for making the estimation. The real cure is to repeat the census at shorter intervals. After a very full account of the present system of registration of births and deaths we come to the question of the registration of sickness, and here we find Dr. Newsholme making the very proper complaint that in so many hospitals classified records have not been regularly kept. What is required, he says, is that all the various reports should be collated on a uniform basis, and that the statistics from the different hospitals should be analysed and published at a central office in London. But in his efforts to obtain a more accurate registration of the sickness existent in the community he would draw to his aid many agencies, and we are afraid that he would if he could draw within his net as notifying agents the whole of the medical profession. This opens out a prospect to which we cannot expect the ordinary practitioner to look forward with unmitigated delight. Many chapters follow which are of very general interest in regard to marriage, fecundity, birth-rates, death-rates, mortality at different ages, influence of climatic and social conditions, density of population, fatality of different diseases, and other subjects. We would strongly urge those who put their faith in crude death-rates—especially medical officers of health at watering places, who, we fear, are often led by their committees to publish roseate views as to the chances of long life within their sanitary areas—to study carefully the chapters describing the corrections to be applied to death-rates before they can be taken to express the truth in regard to the health conditions of any district.

Passing from matters which are of general interest to those of more technical importance, we would express our admiration of the clear and luminous manner in which Dr. Newsholme deals with the question of life tables and of expectation of life. Granted a moderate knowledge of mathematics, and a little familiarity, which practice soon gives, with the uses of tables of logarithms, the whole becomes clear.

Take the book as a whole, it is a most reliable guide to the subject on which it treats, laying down with great exactitude the proper methods in accordance with which the science of numbers may be applied to facts concerning the life history of communities, so as to give true and intelligible results. But it is something more, for even to those who, either from laziness or from ignorance of the methods involved, are quite willing to take Dr. Newsholme's word for all the details; the mere data and the deductions from them form a fact-heap of wide extent and of curious interest.

### NOTES ON BOOKS.

The Art Portfolio for this month contains an admirable reproduction of the well-known picture, "The Knight Errant," by the late Sir J. E. Millais, the original of which is in the Tate Gallery.

Our Baby, by Mrs. Langton Hewer (J. Wright and Co., price 1s. 6d.), is a sixth edition of a very useful little book. It is written for "mothers and nurses," and contains much information and good advice, among which we note that "every mother should possess a pair of scales sufficiently large to weigh baby." If babies were weighed regularly early warning would be given of much disease.

Pasteur—Hydrophobia; and Serotherapy—Diphtheria, Tetanus, Plague, Tuberculosis, Pneumonia, by Dr. A. Lutaud. This is the title on the outer cover of a little sixpenny pamphlet issued by the Anti-Vivisection Society. Inside it is called simply "Hydrophobia and Serumtherapy," but its larger title is the more useful, as showing how many are the diseases which scientific men are endeavouring to cure, and which we suppose the Anti-Vivisection Society prefers to see continue in our midst.

On the Decline of Scurvy Afloat is a very striking little pamphlet, by Surgeon C. March Beadnell, R.N., descriptive of the ravages of scurvy in old days and its remarkable diminution of late years.

The first number of the South Coast Quarterly, of touring and topography, published by Percy Lindley, 30, Fleet Street, contains "The Romance of Industrial Sussex," by Joseph Hatton; papers on Crowborough Hill, Littlehampton, and Leith Hill; and is supplemented by some admirable illustrations of features of interest in the district.

